# Non-operative management for penetrating splenic trauma: how far can we go to save splenic function?

**DOI:** 10.1186/s13017-017-0144-3

**Published:** 2017-07-25

**Authors:** Roy Spijkerman, Michel Paul Johan Teuben, Fatima Hoosain, Liezel Phyllis Taylor, Timothy Craig Hardcastle, Taco Johan Blokhuis, Brian Leigh Warren, Luke Petrus Hendrikus Leenen

**Affiliations:** 1 Department of Trauma, University Medical Centre Utrecht, Heidelberglaan 100, 3584CX Utrecht, The Netherlands; 2 Department of Trauma, Tygerberg Hospital (University of Stellenbosch), Francie van Zijl Avenue, Cape Town, 7505 South Africa; 3 Department of Trauma, Inkosi Albert Luthuli Central Hospital (University of Kwazulu-Natal), 800 Bellair Road, Durban, 4091 South Africa

**Keywords:** Spleen, Penetrating, Non-operative, Trauma, Gunshot wound, Stab wound, Mortality

## Abstract

**Background:**

Selective non-operative management (NOM) for the treatment of blunt splenic trauma is safe. Currently, the feasibility of selective NOM for penetrating splenic injury (PSI) is unclear. Unfortunately, little is known about the success rate of spleen-preserving surgical procedures. The aim of this study was to investigate the outcome of selective NOM for penetrating splenic injuries.

**Methods:**

A dual-centre study is performed in two level-one trauma centres. All identified patients treated for PSI were identified. Patients were grouped based on the treatment they received. Group one consisted of splenectomised patients, the second group included patients treated by a spleen-preserving surgical intervention, and group three included those patients who were treated by NOM.

**Results:**

A total of 118 patients with a median age of 27 and a median ISS of 25 (interquartile range (IQR) 16–34) were included. Ninety-six patients required operative intervention, of whom 45 underwent a total splenectomy and 51 underwent spleen-preserving surgical procedures. Furthermore, 22 patients (12 stab wounds and 10 gunshot wounds) were treated by NOM. There were several anticipated significant differences in the baseline encountered. The median hospitalization time was 8 (5–12) days, with no significant differences between the groups. The splenectomy group had significantly more intensive care unit (ICU) days (2(0–6) vs. 0(0–1)) and ventilation days (1(0–3) vs. 0(0–0)) compared to the NOM group. Mortality was only noted in the splenectomy group.

**Conclusions:**

Spleen-preserving surgical therapy for PSI is a feasible treatment modality and is not associated with increased mortality. Moreover, a select group of patients can be treated without any surgical intervention at all.

## Background

The spleen plays an important role in the immune system, and asplenia is associated with a lifelong increased risk of severe infectious diseases [1, 2]. Currently, splenic injuries are therefore preferably treated in a way that splenic function can be preserved [3, 4]. It has been shown that over 80% of blunt injuries to the spleen can be treated by non-operative management (NOM) [3, 5]. Moreover, when laparotomy is indicated, there are several surgical options to treat splenic injuries besides a total splenectomy [6, 7]. These spleen-saving procedures have been shown to be safe and effective in saving the immunologic function of the spleen in blunt splenic trauma [8].

It is unclear, however, whether selective NOM and spleen-preserving surgery is suitable for the treatment of penetrating splenic injuries as well. Non-operative and spleen-preserving surgery for the treatment of penetrating solid intra-abdominal organs is becoming more common in large trauma centres that frequently deal with penetrating trauma [9–11]. Most studies that address the feasibility of NOM focus on the injured liver or kidney [12, 13]. As splenic injuries are relatively rare, most other studies only analyse pooled data from all intra-abdominal organs (including the spleen) [9–11]. In order to investigate the feasibility of selective NOM and spleen-preserving surgery for the spleen, it is essential not to consider the injured spleen comparable to liver and kidney injuries. The clinical course of splenic injuries is considerably different from other solid intra-abdominal organs as splenic injuries are notorious for the risk of delayed bleeding [14]. When the spleen is injured, there is a high chance of concurrent intra-abdominal solid and hollow organ injuries as well as thoracic and diaphragmatic injuries [15].

Little is known about the feasibility and safety of NOM and spleen-preserving surgery for the treatment of PSI (penetrating splenic injury). One recent single-centre study from Berg et al. indicated that NOM can be utilized in a select group of patients with penetrating splenic trauma [15]. In our institutions, more liberal inclusion criteria for selective non-operative management are used; therefore, we aimed to explore the safety of our protocols, in which we push spleen-saving therapy to the limits.

## Methods

We performed a dual-centre study in two level-one trauma centres in South-Africa to investigate the feasibility of selective NOM in PSI. We received approval from the Health Research Ethics Committee (HREC) in Cape Town and the Biomedical Research Ethics Committee (BREC) in Durban. From the prospectively composed trauma database in Tygerberg Hospital in Cape Town as well as the Inkosi Albert Luthuli Central Hospital (IALCH) in Durban, we retrospectively identified patients that presented to either institution for the treatment of penetrating splenic injury. The study period in Tygerberg Hospital was between September 1, 2010, and September 1, 2014, while we included all patients presented to IALCH between April 1, 2007, and April 1, 2014. All patients with a splenic injury presenting to the IALCH were identified from the institutional trauma registry (UKZN BREC BE207–09). We identified the patients at Tygerberg Hospital by reviewing a maintained operation logbook as well as the radiology database (HREC S14/02/046). All patients above the age of 14 were included. For the purpose of the study, we excluded patients who died in the emergency department before diagnostic work-up was completed.

### Study group characteristics

Documented data included patient demographics: age in years, gender, systolic blood pressure (SBP) in millimetre of mercury, pulse rate (PR) in beats per minute, Glasgow Coma Score (GCS), serum haemoglobin (Hb) in grams per decilitre, serum haematocrit (Ht) in L/L, thrombocyte count in ×10^9^/L, Abbreviated Injury Scale (AIS) [16] and Injury Severity Score (ISS) [17]. We further documented the specific underlying mechanism of injury, thereby distinguishing stab wounds (SW) from gunshot wounds (GSW).

### Imaging

Computed tomography (CT)-scan reports were documented and used for this study. All patients that were haemodynamically stable enough were preoperatively scanned by CT. Also, all patients that were selected for NOM underwent a CT-scan.

### Treatment modalities

**Fig. 1:**
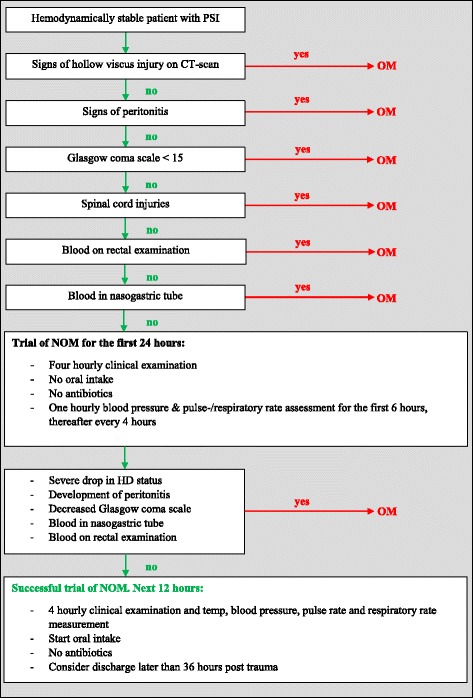
Treatment protocol

Patients were categorized by the type of treatment that they received. Group I consisted of patients treated by a total splenectomy, patients that underwent a spleen-preserving surgical procedure were included in group II, and patients treated by NOM were analysed as group III. Spleen-preserving surgery is a procedure where the bleeding from the spleen was either stopped by the use of sutures or by the use of haemostatic techniques, such as the application of Surgicel® (Ethicon, Johannesburg). Patients that were treated by NOM underwent a successful trial of NOM. In order to make a trial of NOM successful, we created new treatment guidelines (Fig. 1). Not all the patients included in our study period followed this protocol, but we have started using it currently.

We suggest a trial of NOM in patients with penetrating splenic injury without a strict indication (such as HVI) for operative management (OM). We utilize the following other exclusion criteria for NOM: decreased level of consciousness, spinal cord injuries, blood in nasogastric tube, and blood on rectal examination. All patients have to undergo CT scanning to identify concurrent HVI and to grade concurrent intra-abdominal injuries. A trial of NOM includes a strict observation period of 24 h with serial clinical examination and temperature every 4 h, no oral intake, no antibiotics, one hourly blood pressure and pulse/respiratory rate measurements for the first 6 h and thereafter every 4 h. If the first 24 h are uneventful, it is recommended to give a trial of feeding and perform clinical examination every 4 h during the next 12 h without antibiotics. If there are signs of neurological problems, signs that indicate HVI or signs of haemodynamic instability consider operative management (OM). It is recommended to discharge patients no earlier than 36 h after admission.

### Outcomes

The primary outcome was mortality. The secondary outcomes were post-operative complications, mechanical ventilation days, length of hospital stay (LOS) and length of intensive care unit (ICU) stay. We also compared the outcome of patients sustaining gunshot wounds with those that sustained stab wounds. Splenic-AIS and AIS of associated injuries were determined by using the 1998 version of the Abbreviated Injury Scale.

### Statistical analysis

Statistical analysis was performed using SPSS for Windows 20.0 (IBM, Chicago, Illinois). Differences between groups were calculated with Fisher’s exact test and chi-square test for ordinal data and two-tailed *t* test and Mann-Whitney *U* test for continuous data. *P* values less than 0.05 were considered significant.

## Results

For the purpose of this study, we identified, during a 4-year period at the Tygerberg Hospital and a 6-year period at the Inkosi Albert Luthuli Central Hospital, all patients with penetrating splenic injuries.

### Study group characteristics

A total of 118 patients (109 (92%) male and 9 (8%) female) with a median (interquartile range (IQR)) age of 27 (20–32) presented to the emergency departments. On admission, they had a median (IQR) systolic blood pressure of 122 (105–136), a pulse rate of 94 (80–113) beats per minute and a Glasgow Coma Scale-score of 15 (15–15). Nineteen patients (16%) had an altered mental state (GCS < 15). Fifty-three patients (45%) were admitted for the treatment of stab wound injuries, whereas 65 patients (55%) sustained gunshot injuries. The median (IQR) Abbreviated Injury Scale of the encountered splenic lesions was 3 (3–4). Seventy-eight individuals (66%) had a splenic AIS <4, while 40 patients (33%) were diagnosed with a grade 4 or 5 splenic injury. The patients had a median total Injury Severity Score of 25 (16–34).

### Comparison of baseline characteristics

A comparison of baseline characteristics of the populations is shown in Table 1. As expected, there were significant differences in age, systolic blood pressure, pulse rate, GCS and thrombocyte count between the splenectomy and the spleen-preserving surgical therapy group. Furthermore, median (IQR) Abbreviated Injury Score (4 (3–5) vs. 2 (2–3)) and Injury Severity Score (25 (19–16) vs. 18 (13–25)) were both significantly higher in patients who underwent a splenectomy. In the splenectomy group, 20 patients (44%) had a splenic-AIS <3, a total of six patients (13%) had a splenic-AIS of 4 and 19 patients (43%) were found with an AIS of 5. In the spleen-preserving surgical treatment group, 43 patients (84%) had a splenic-AIS <4, three patients (6%) had AIS of 4, and five patients (10%) were diagnosed with an AIS of 5.

**Table 1:** Comparison of baseline characteristics

	Group 1: Splenectomy (*n* = 45)	Group 2: Spleen-preserving surgical therapy (*n* = 51)	Group 3: Non-operative management (*n* = 22)
Age (years)	29 (22–34)^a^	26 (19–30)^a^	26 (20–32)
Gender (M/F)	42/3	47/4	20/2
SBP (mmHg)	117 (94–137)^a^	129 (115–141)^a^ ^,Φ^	117 (105–124)^Φ^
Pulse rate (bpm)	103 (82–124)^a^	89 (74–108)^a^	98 (83–106)
GCS	15 (14–15)^†^	15 (15–15)^†^	15 (15–15)
Serum Hb (g/dL)	11 (9.2–12.7)	11.5 (9.7–13.4)	11.8 (10–12.8)
Thrombocytes count (×10^9^/L)	212 (124–290)^a^^,‡^	274 (208–319)^a^	281 (208–391)^‡^
AIS spleen	4 (3–5)^†^	2 (2–3)^†,∞^	3 (2–4)^∞^
ISS	25 (19–36)^a^	18 (13–25)^a^	27 (18–41)

All variables are in median (IQR)

*SBP* systolic blood pressure, *bpm* beats per minute, *GCS* Glasgow Coma Score, *Hb* haemoglobin, *Ht* haematocrit, *ISS* Injury Severity Score, *AIS* Abbreviated Injury Score

Group 1 vs. group 2 with *p* < 0.05 by ^a^ *t* test and ^†^Mann-Whitney *U* test

Group 2 vs. group 3 with *p* < 0.05 by ^∞^ *t* test and ^Φ^Mann-Whitney *U* test

Group 1 vs. group 3 with *p* < 0.05 by ^‡^ *t* test

Non-operatively managed patients had a significantly lower systolic blood pressure (117 (105–124) vs. 129 (115–141)) and a significantly higher splenic-AIS (3 (2–4) vs. 2 (2–3)) compared to patients selected for spleen-preserving surgical therapy. Fifteen out of 22 patients (68%) that were selected for non-operative management had an AIS <3, two patients (9%) had an AIS of 4, and five patients (23%) had an AIS of 5.

### Mechanism of injury

We compared the characteristics, management and outcome of patients with either gunshot or stab wound injuries. As anticipated, the AIS of the splenic injury (3 (2–5) vs. 2 (2–3)) as well as total ISS (25 (18–41) vs. 18 (13–25)) were significantly higher in patients suffering from gunshot wounds.

A comparison between the management and outcome of stab wounds and gunshot wounds is shown in Table 2. Splenectomy was relatively more frequently performed in the patients suffering from gunshot wounds (SW = 10/53 (19%) vs. GSW = 35/65 (54%)). The amount of patients managed non-operatively does not significantly differ between stab wounds and gunshot wounds (SW = 12/53 (23%) vs. GSW = 10/65 (15%)). The total number of complications is significantly higher in patients with gunshot wounds (50 vs. 6). The number of ventilation days (1 (0–3) vs. 0 (0–0)), the number of days in the ICU (3 (1–8) vs. 0 (0–0)) and hospitalization days (9 (6–18) vs. 6 (5–9)) are significantly higher in patients suffering from gunshot wounds then those treated for stab wound injuries. Furthermore, fatalities were only seen in the patients with gunshot injuries (*N* = 7).

**Table 2:** Mechanism of injury

	Stab wounds *n = 53*	Gunshot wounds *n = 65*
Total splenectomy	10	35
Spleen-preserving therapy	31	20
Non-operative management	12	10
Total number of complications	6^a^	50^a^
No. of patients with complications	5	27
Ventilation days	0 (0–0)^∞^	1 (0–3)^∞^
ICU stay (days)	0 (0–0)^∞^	3 (1–8)^∞^
Length of hospital stay (days)	6 (5–9)^∞^	9 (6–18)^∞^
Mortality	0^†^	7^†^

All variables are in median (IQR). All frequencies are in absolute number

*ICU* intensive care unit

*p* < 0.05 by ^a^chi-square test, ^†^Fisher’s exact test and ^∞^Mann-Whitney *U* test

### Associated injuries

The associated injuries found in our distinct study groups are shown in Table 3. Concomitant solid intra-abdominal organ injuries were encountered in all groups. Left kidney injuries are the most frequently associated abdominal injuries, a total of 48 out of 118 patients (41%) were diagnosed with this injury. Stomach and colon injury are the two most frequently seen hollow viscus injuries (HVIs). There were no HVIs found in the patients managed non-operatively. Thirty-two out of 45 patients (71%) from the splenectomy group had a concurrent diaphragm injury, while 35 out of 51 patients (69%) from the patients treated by a spleen-preserving surgical intervention had a diaphragmatic lesion. The most common injured extra abdominal organ is the lung (93 out of 118 patients (79%)), most frequently due to a pneumothorax. Furthermore, two of the splenectomised patients (4%) had a concurrent cardiac injury.

**Table 3:** Associated injuries

	Group 1: Splenectomy (*n* = 45)	Group 2: Spleen-preserving surgical therapy (*n* = 51)	Group 3: Non-operative management (*n* = 22)
Abdominal solid organ injuries			
Kidney	29	20	10
Liver	18	10	3
Pancreas	16	7	1
Abdominal hollow organ injuries			
Stomach	22	15	0
Colon	16	12	0
Small bowel	6	7	0
Duodenum	1	0	0
Extra-abdominal injuries			
Lung	32	43	18
Diaphragm	33	35	5
Spine	7	6	7
Craniocerebral	2	1	4
Heart	2	0	0
Neck	2	1	0
Maxillofacial	1	0	1
Ureter	1	0	0

### Treatment modalities

**Fig. 2:**
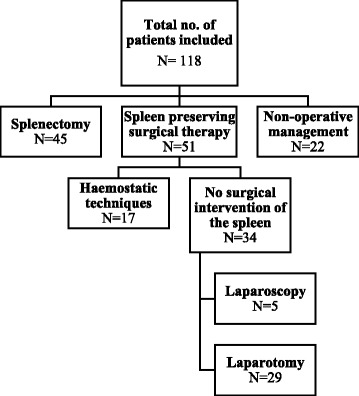
Flowchart

A total of 96 out of 118 patients (81%) required immediate surgical intervention, of whom 91 patients (77%) underwent a laparotomy and five patients (4%) underwent a diagnostic laparoscopy. Forty-five of the 118 patients (38%) were splenectomised. One of the splenectomised patients was initially selected for non-operative management; however, during an electively executed diaphragmatic repair procedure, the spleen started bleeding again after manipulation. As haemostatic techniques were unable to stop the blood loss, a splenectomy was inevitable. A total of 51 of the 118 patients (43%) were treated by spleen-preserving surgical treatment. There were several indications for an operative intervention without the need for splenectomy. We can divide into two big groups. The first group included patients that needed emergency surgery for haemodynamic instability, but where the main source of bleeding was mostly a different organ/vessel. In 17 of these 51 patients (33%), the spleen was bleeding, but the bleeding could be stopped by haemostatic agents. The second big group included patients that received an operative intervention for delayed peritonitis or where the patient was operated upon for the evaluation of diaphragm injuries. Thirty-four out of 51 patients (66%) underwent an operative intervention for the treatment of their abdominal injuries without the need to actively treat the splenic injury. Of this group, 29 of the 34 patients (85%) underwent a laparotomy and five patients (15%) underwent a laparoscopy for repair of their diaphragm injury. Furthermore, 22 of the 118 patients (19%) with splenic injuries were treated by non-operative management. The treatment modalities that were used in our patients are illustrated in Fig. 2.

### Morbidity and mortality

A comparison of outcome measurements in patients treated by different treatment modalities is demonstrated in Table 4. The number of complications did not differ in terms of statistical significance between groups. Nevertheless, the splenectomy group had a significantly higher number of people suffering from complications than the spleen-preserving group (19/45 (42%) vs. 9/51 (18%)). Mechanical ventilation days (1 (0–3) vs. 0 (0–0)) and duration of ICU stay (2 (0–6) vs. 0 (0–1)) were significantly longer in splenectomised patients compared to non-operated patients. All the complications are listed in Table 5. The majority of our complications were found in the splenectomy group. The most prevalent complications in our study were intra-abdominal collections which complicated the clinical course of patients 16 times (14%). Six patients (5%) had pneumonia, and 10 patients (8%) were diagnosed with sepsis. A total of three patients (3%) developed multi-organ dysfunction syndrome (MODS). There was a statistically significant difference in mortality between the splenectomy group and the spleen-preserving surgical treatment group. There was no mortality in patients selected for spleen-preserving surgical procedures and in the non-operative group, while seven of the 45 patients (16%) from the splenectomy group died during hospitalization. Three of the 45 patients (7%) died several hours after operative intervention due to massive and ongoing blood loss, while four other patients (9%) died later mainly due to MODS.

**Table 4:** Comparison of outcome between treatment groups

	Group 1: Splenectomy (*n* = 45)	Group 2: Spleen-preserving surgical therapy (*n* = 51)	Group 3: Non-operative management (*n* = 22)
Total number of complications	30	21	5
No. of patients with complications	19^a^	9^a^	4
Ventilation days	1 (0–3)^∞^	0 (0–1)	0 (0–0)^∞^
ICU stay (days)	2 (0–6)^∞^	0 (0–3)	0 (0–1)^∞^
Length of hospital stay (days)	8 (7–12)	7 (5–12)	8 (5–15)
Mortality	7^†^	0^†^	0

All variables are in median (IQR). All frequencies are in absolute number

*ICU* intensive care unit

Group 1 vs. group 2 with *p* < 0.05 by ^a^chi-square test and ^†^Fisher’s exact test

Group 1 vs. group 3 with *p* < 0.05 by ^*∞*^Mann-Whitney *U* test

**Table 5:** Post-operative complications

	Group 1: Splenectomy (*n* = 45)	Group 2: Spleen-preserving surgical therapy (*n* = 51)	Group 3: Non-operative management (*n* = 22)
Intra-abdominal collections	7	7	2
Perisplenic	5	1	1
Other location	3	6	1
Sepsis	6	3	1
Pneumonia	2	2	2
Ileus	3	–	–
MODS	3	–	–
Wound infection	1	2	–
Ongoing bleeding	2	–	–
Re-bleed	2	–	–
Subglottic stenosis	–	2	–
Renal pseudo aneurysm	1	–	–
Lung empyema	1	1	–
Upper GI bleed	1	–	–
Enterocutaneous fistula	1	–	–
Exudative pleural adhesions	–	1	–
Extra-abdominal collections	–	1	–
Infected urinoma	–	1	–
Urinoma	–	1	–

All frequencies are in absolute number

*MODS* multi-organ dysfunction syndrome, *GI* gastrointestinal

## Discussion

In our study, 22 out of 118 patients (19%) with penetrating splenic injuries were successfully treated by non-operative management; therefore, we can conclude that NOM is feasible in a selected group of patients. More than half of the patients (51/98) with PSI that required emergency laparotomy were successfully treated by spleen-preserving surgical procedures, and therefore, we conclude that spleen-saving surgery is a safe alternative to total splenectomy, even for penetrating trauma. This study also noted that both NOM and spleen-saving surgery can be applied in both stab and gunshot injuries. Thus, based on our findings, we recommend considering non-operative therapy in all haemodynamically stable patients with penetrating splenic injuries, without concurrent injuries that need an operation, as a feasible alternative to routine operative exploration in appropriate high-level trauma care facilities.

### Stab wounds

We found that splenic stab wound injuries in patients, without concurrent intra-abdominal hollow viscus organ injuries and haemodynamic instability, can be successfully treated by non-operative management. Complication rates are low, and delayed re-bleeds are rare as only one non-operatively treated patient required splenectomy for the treatment of secondary bleeding. Furthermore, it cannot be excluded that this single case of delayed splenic bleeding was not a result of iatrogenic injury, as it occurred during an elective diaphragm repair operation. Hence, we would recommend consideration of non-operative management in all haemodynamically stable patients with splenic injury caused by stabbing. Nevertheless, due to the high number of associated concurrent hollow organ injuries (57 out of 118 had at least one concurrent HVI), we strongly advise to routinely perform computed tomography scanning and good clinical review in all patients with splenic stab wounds selected for non-operative management.

### Gunshot wounds

Given the high complication rates in patients with gunshot injuries, we believe that our study does not provide sufficient evidence to recommend selective NOM for patients with splenic gunshot injuries. According to our univariate analysis, it appears that the mechanism of injury has to be considered as a key predictive factor for morbidity. Patients non-operatively treated for gunshot injuries had significantly more complications than those treated for stab wound injuries. This difference can be explained by the increased number of associated hollow and solid intra-abdominal, as well as thoracic, injuries. Additionally, both ICU stay and hospitalization time were statistically significantly prolonged in patients treated for gunshot injuries. We do not recommend the routine use of NOM for patients suffering from gunshot injuries. These findings are in line with a systematic review performed by Singh and Hardcastle, where they concluded that NOM is feasible for GSW, but it can not be used routinely [18]. More studies should be performed in order to define specific criteria for adequate patient selection. Until these studies are executed, we feel safe to select stable patients with minor gunshot injuries to the spleen and normal mental status for a trial of non-operative management. In our study, a total of 10 patients with gunshot injuries to the spleen were successfully treated without surgical intervention. A key pre-requisite for these guidelines is the availability of decent monitoring facilities and adequately trained staff.

### Haemodynamically unstable patients

When a haemodynamically unstable patient with penetrating splenic injury presents to the emergency department, a trial of NOM is not possible. Emergency laparotomy is indicated. However, in contrast to haemodynamically unstable patients with blunt splenic trauma, patients with penetrating injuries have frequently less concomitant foci of blood loss. This diminishes the necessity of a fast splenectomy aimed to reduce crucial operation time in the damage control setting. We opt to utilize an alternative surgical strategy in which the spleen is not routinely and directly taken out, but packed or treated by local haemostatic techniques and then left untouched during the rest of the inspection of the abdomen. In accordance with our treatment guidelines, attempts to preserve splenic function are the preferred treatment for penetrating splenic injuries. In more than half of our patients, we managed to preserve the spleen by this approach.

### Reflection of the literature

Our results of stab and gunshot injuries are in line with the literature. A retrospective study performed by Berg et al. concluded that a select group of patients without haemodynamic instability, peritonitis and radiologic evidence of hollow organ injuries might be adequate candidates for a trial of NOM [15]. They further suggested the need to gather multi-centre data in order to define more precisely the selection criteria for NOM. Their study was executed in a level one trauma centre in the USA and investigated 238 cases of PSI of whom eventually less than 10% (24 patients) were successfully treated by non-operative treatment. In fact, 10 of these 24 patients cannot, however, be considered as proper cases of NOM, since those patients underwent minimally invasive surgery for the evaluation of their abdominal injuries. According to our more strictly defined definitions of NOM in their study, only 2.4% of patients were successfully treated by non-operative therapy. We treated approximately 20% of patients with PSI successfully by NOM. The fraction of patients that is suitable for NOM seems to be almost 10 times larger than that suggested by Berg et al. [15].

In a study from Clancy et al., 57 out of 197 patients with penetrating splenic injuries were initially selected for non-operative therapy; however, the failure rate of NOM is not documented [19]. In other series from Pachter et al. [20], Demetriades et al. [11] and Kaseje et al. [21], relatively less patients with PSI were selected for NOM (respectively 6/43, 3/28 and 5/25). In the study from Demetriades et al., NOM failed in two out of three patients while there was no failure documented in the other studies [11]. Despite the absence of failure rates of NOM, our study is the first to describe successful non-operative treatment of about 20% of patients admitted to level one trauma centres for the treatment of PSI.

### Key points and limitations

The key points of this study are that we were able to combine data from two large South African trauma centres and that patients in both institutions were treated according to the same treatment guidelines. Furthermore, this study is unique in enabling us to describe the natural course of penetrating splenic injuries without immediate treatment. We were (unwillingly) forced to push non-operative management to its limits. Hence, given our results, we have probably not reached these limits yet, at least not for stab wound injuries of the spleen.

The main limitation of this study is the retrospective design. However, due to a strictly maintained trauma and radiology data registry, we were able to identify a large number of patients and we had unlimited excess to all patient charts and laboratory results. So the chance of under registration of findings is considered to be very low. Another disadvantage of the study is the fact that we had to exclude several patients as they were transferred from referring hospitals to our level one trauma centres. Also due to the retrospective design, the baseline characteristics show some significant differences between the treatment groups. Most of the differences can be attributed to the fact that patients that need surgical intervention are in a different clinical state than patients that can be selected for NOM. However, there might be some part of selection bias due to these baseline differences. There also is a risk of selection bias due to the situation in the hospitals in South Africa; it is different to the situation in hospitals in the western world. Doctors have to deal with limited resources, and different decisions are made regarding treatment modalities.

## Conclusions

In conclusion, our study is the first to show that non-operative management for penetrating splenic trauma is a feasible alternative in selected patients. Given the large number of complications in patients with gunshot wounds to the spleen, we recommend to be reluctant with applying NOM in this group. However, patients with stab wounds seem to be feasible candidates for non-operative therapy. Due to the high prevalence of concurrent hollow viscus organ injuries in patients with PSI, we consider adequate computed tomography imaging as a key pre-requisite for NOM. Furthermore, we believe that spleen-saving techniques must have an important place in treatment algorithms of emergency surgery.

## Abbreviations

AIS: Abbreviated Injury Score
BPM: Beats per minute)
CT: Computed tomography
GCS: Glasgow coma scale
GWS: Gunshot wound
Hb: Haemoglobin
Ht: Haematocrit
HVI: Hollow viscus unjury
IALCH: Inkosi Albert Luthuli Central Hospital
ICU: Intensive care unit
IQR: Interquartile range
ISS: Injury Severity Score
LOS: Length of hospital stay
MODS: Multi-organ dysfunction syndrome
NOM: Non-operative management
OM: Operative management
PR: Pulse rate
PSI: Penetrating splenic injury
SBP: Systolic blood pressure
SW: Stab wound
